# Heading the ball, why does the neurologist need to know about it?

**DOI:** 10.1055/s-0045-1801864

**Published:** 2025-03-10

**Authors:** Renato Anghinah

**Affiliations:** 1 Universidade de São Paulo, Faculdade de Medicina, Hospital das Clínicas, São Paulo SP, Brazil.

The new approach concerns head trauma and sport concussion, and these consequences, as chronic traumatic encephalopathy started to be on highlights in those last two decades. The increasing number of cases of concussion in sports like soccer brings us general neurologists a new challenge, understanding what the consequences of sports head hits are and what we can do to mitigate their consequences.

The patients could be on the field, in the office, or in the hospital; whether caring for acute or chronic neurologic diseases, and whether caring for individuals who participate in organized sports or just occasional exercise, each clinical neurologist will encounter patients whose neurologic disorders either are a consequence of or impact upon participation in an athletic activity. 1

In this issue the article titled “Cognitive functioning and soccer heading: one-year longitudinal assessment among professional players”, authored by Giovanni Batista Palma, Mariana Drummond Martins Lima, Ana Carolina Oliveira Rodrigues, Clarisse Vasconcelos Friedlaender, Celso Furtado de Azevedo Filho, Rodrigo Campos Pace Lasmar, and Paulo Caramelli, provide us with the information to take one more step forward to understand how soccer heading ball could be a risk factor for the athletes and if sports related could contribute with brain damage. 2

The authors followed for a year a professional soccer team, so that they studied 22 soccer players from a main division of Brazilian soccer championship, testing all players before the beginning of championship and running a second evaluation on the end of the season.

This is something very difficult to do in Brazilian soccer teams. The authors found the soccer players outperformed the controls at baseline on some measures and outperformed controls when baselines were compared to end of season data.

The real impact of the ball from soccer players is not conclusive but opens an opportunity to conclude that in one year of follow-up, there are not big changes in cognition of soccer players and the continuity of follow-up could be essential to observe if the time of exposition could be a risk for them.

Another contribution concerns performance, where the athletes showed a better result compared to controls, what could mean that the practice of attention and motor skills could be a relevant point of study and could be a contribution for rehabilitation services.
